# Disrupted Cerebellar-Default Mode Network Functional Connectivity in Major Depressive Disorder With Gastrointestinal Symptoms

**DOI:** 10.3389/fncel.2022.833592

**Published:** 2022-03-03

**Authors:** Yudan Ding, Yangpan Ou, Haohao Yan, Xiaoya Fu, Meiqi Yan, Huabing Li, Feng Liu, Wenbin Guo

**Affiliations:** ^1^Department of Psychiatry, National Clinical Research Center for Mental Disorders, The Second Xiangya Hospital of Central South University, Changsha, China; ^2^Department of Radiology, The Second Xiangya Hospital of Central South University, Changsha, China; ^3^Hunan Key Laboratory of Children’s Psychological Development and Brain Cognitive Science, Changsha, China; ^4^Department of Radiology, Tianjin Medical University General Hospital, Tianjin, China; ^5^Department of Psychiatry, The Third People’s Hospital of Foshan, Foshan, China

**Keywords:** major depressive disorder, gastrointestinal symptoms, functional magnetic resonance imaging, cerebellum, default mode network

## Abstract

Gastrointestinal (GI) symptoms are one of the common somatic symptoms presented in patients with major depressive disorder (MDD). Higher frequency of GI symptoms and higher GI symptom burden were linked to greater depression severity and increased risk of suicide ideation. However, few studies have explored the underlying mechanisms of GI symptoms in MDD. Based on previous studies, the cerebellar-DMN circuits may play a potentially critical role in GI symptoms comorbid with depression. Fifty-two first-episode drug-naive patients with MDD (35 with GI symptoms and 17 without GI symptoms) and 28 matched healthy controls were recruited in the current study and underwent resting-state functional magnetic resonance imaging scan. Cerebellar seed-based functional connectivity maps were established. Relative to depressed patients without GI symptoms, significantly increased cerebellar-anterior default mode network (DMN) connectivities were found in those with GI symptoms. Both increased and decreased functional connectivities were found between cerebellum and posterior DMN in patients with GI symptoms compared with those without GI symptoms and healthy controls. Moreover, the right Crus I - right superior temporal gyrus connectivity value was related to severity of GI symptoms and depression in all patients with MDD. The support vector machine analysis demonstrated a satisfactory classification accuracy (89%) of the disrupted cerebellar-DMN connectivities for correctly identifying MDD patients with GI symptoms. These results revealed the possible neural mechanisms for the involvement of cerebellar-DMN circuits in GI symptoms co-occurred with MDD.

## Introduction

Major depressive disorder (MDD) is one of the most common and debilitating psychiatric disorders worldwide characterized by heterogeneous symptoms and highly variable course trajectories (Fried and Nesse, 2015). Besides a pervasive and persistent depressed mood and loss of interest or pleasure, seven other symptoms are listed in the Diagnostic and Statistical Manual of Mental Disorders, fifth edition (DSM-5), including weight or appetite changes, sleep disturbances, psychomotor agitation or retardation, physical symptoms, cognitive disturbances, feelings of inappropriate worthlessness, helplessness or hopelessness, and suicidal thoughts or suicide attempt (American Psychiatric Association, 2013). Notably, somatic symptoms often accompany with depression and have a prevalence comparable to that of psychological symptoms (Simon et al., 1999). Many patients with MDD visit primary care physicians with somatic symptoms as the chief complaint. According to an international study across 14 countries on five continents, 45–95% patients with MDD visiting primary care settings only reported somatic symptoms (Simon et al., 1999). It is well-acknowledged that Chinese individuals tend to express somatic symptoms and accept a physical disease diagnosis rather than admitting to having mental illness (Parker et al., 2001, 2005; Zhao et al., 2018), which hinders early diagnosis and appropriate treatment and consequently results in aggravation of symptoms and even treatment resistance (Smith, 2014; Dunlop et al., 2020). The presence of somatic symptoms is supposed to have associations with more severe depressive symptoms, worse health-related quality of life, and lower remission rates in patients with MDD in the psychiatric care settings (Lee et al., 2009; Novick et al., 2013; Woo et al., 2014). Evidence indicated that improvements of painful physical symptoms after antidepressant treatment were associated with higher remission rates (Fava et al., 2004). Somatic symptoms were also found to be significant risk factors for current suicidal ideation in depressed patients (Jeon et al., 2016; Fang et al., 2019). Given the important role of somatic symptoms in clinical decision-making and prognosis of MDD, it is essential to clarify the clinical characteristics and pathophysiology of varies somatic symptoms in MDD.

Gastrointestinal (GI) symptoms, such as nausea, vomiting and gastric distention, are one of the common somatic symptoms presented in patients with MDD. Approximately 30–70% patients with MDD had concomitant GI symptoms (Zhao et al., 2018; Huang et al., 2021). Consistent with the abovementioned findings from somatic symptoms, higher frequency of GI symptoms and higher GI symptom burden were linked to greater depressive severity and increased risk of suicide ideation (Soderquist et al., 2020; Huang et al., 2021). Although a lot of efforts have been made to explore the underlying mechanisms of somatic symptoms in MDD, few focuses have been put on GI symptoms. Evidence from several neuroimaging studies indicated that brain structural and functional changes may contribute to the neuropathology of GI symptoms in patients with MDD (Liu P. et al., 2019; Liu et al., 2020; Liu Y. et al., 2021; Yan et al., 2021). For example, reduced gray matter volume and regional homogeneity were found in the bilateral middle frontal gyrus, left precentral gyrus, and right superior frontal gyrus in MDD patients with GI symptoms, whereas enhanced regional homogeneity was found in the left temporal gyrus (Liu et al., 2020). In another study, decreased gray matter volume were mainly observed in frontal and occipital cortex, whereas increased gray matter volume was observed in the limbic system (Liu P. et al., 2019). Our team found abnormal brain function mainly in the default mode network (DMN) and cerebellum posterior lobe in MDD patients with GI symptoms using whole brain-based method (Liu Y. et al., 2021; Yan et al., 2021).

It is well-known that affective disorders are common comorbidities of functional gastrointestinal disorders (FGID). Co-occurrence of depression was found in about 30% patients with FGID in primary care settings and even higher in tertiary care (Van Oudenhove et al., 2016). Interestingly, researches on FGID have greatly progressed, revealing that brain intrinsic networks were altered in this group of diseases and theses abnormalities may be potential biomarkers to differentiate these diseases from healthy controls (Kano et al., 2018). For instance, decreased global efficiency (Qi et al., 2016a) and decreased amplitude of low-frequency fluctuations (Qi et al., 2016b) were found in the DMN in patients with irritable bowel syndrome. Altered brain activities were also reported in the cerebellum and multiple regions in functional dyspepsia, and these resting-state patterns could discriminate between patients and controls with a high level of accuracy (Liu et al., 2013; Zhou et al., 2013). Thus, the cerebellar-DMN circuits may play a potentially critical role in GI symptoms comorbid with depression.

Extending beyond the motor aspect, cerebellar contributions to cognition and affect have attracted numerous interests over the past decades. Neuroimaging studies revealed a complex configuration of cognitive/affective topographic organization in the cerebellum (Depping et al., 2018). Generally, the lobule IX and Crus I are considered as the DMN representation (Habas et al., 2009; Krienen and Buckner, 2009; O’Reilly et al., 2010; Buckner et al., 2011; Bernard et al., 2012). Structural and functional abnormalities of cerebellar the DMN components have been repeatedly reported in patients with MDD. Aberrant lobule IX volume was observed in patients with recurrent depression regardless of acute or remitted stage (Depping et al., 2016). Different research teams reported reduced cerebellar-cerebral (including the DMN) connectivity in patients with MDD (Liu et al., 2012; Guo et al., 2013). However, they either did not subtype patients according to different clinical, neuropsychological, or neurophysiological features, or did not focusing on GI symptoms.

In the current study, we aimed to explore the intrinsic cerebellar functional connectivity differences across MDD patients with and without GI symptoms and healthy controls using seed-based resting-state functional magnetic resonance imaging (fMRI), and to investigate the possible associations between altered cerebellar functional connectivity and the severity of GI symptoms. Based on previous evidence reviewed above, we hypothesized that there would be disrupted cerebellar-DMN connectivity involved with GI symptoms in patients with MDD. Furthermore, we examined whether the altered cerebellar-DMN connectivities could serve as potential biomarkers for GI symptoms in MDD and reliably discriminate MDD patients with GI features from those without GI symptoms or healthy controls employing support vector machine (SVM) approach.

## Materials and Methods

### Participants

A total of fifty-two first-episode drug-naive patients diagnosed with MDD according to the DSM-5 criteria by professional psychiatrists were recruited from the Second Xiangya Hospital of Central South University, including 35 patients with GI symptoms and 17 patients without GI symptoms. None of the patients had other Axis I or Axis II psychotic disorders. While 28 demographically similar healthy controls without a history or a family history of psychiatric disorders were recruited from the local community. All individuals were 18–55 years old and right-handed. Subjects would be ruled out if they: (1) had acute physical illness (here only included structural or organic diseases, and subjects with FGIDs were not included in the exclusion criteria) or neurological illness, or a history of substance abuse; (2) had a history of brain injury resulting in loss of consciousness; (3) were pregnant or were unable to undergo MRI scans. All the participants provided a written informed consent, and the study was obtained approval from the ethics committee of the Second Xiangya Hospital of Central South University.

Symptom severity was assessed using 17-item Hamilton Rating Scale for Depression (HRSD-17) by a trained clinical psychiatrist. Specifically, items 10, 11, 12, 15, and 17 represent anxiety/somatization factor, item 16 represents weight loss factor, items 2, 3, and 9 represent cognitive disturbance factor, items 1, 7, 8, and 14 represent retardation symptoms, items 4, 5, and 6 represent sleep disturbance, and item 12 represents GI symptoms.

### Magnetic Resonance Imaging Acquisition

Magnetic resonance imaging scanning was conducted on a Siemens 3T scanner (Siemens Medical Systems, Erlangen, Germany). During the scanning, all participants were instructed to keep relax, motionless, and keep eyes closed but not fall asleep. Foam pads and earplugs were used to minimize head motion and reduce scanner noise, respectively. Resting-state fMRI data were acquired using a gradient echo-planar imaging sequence with the following parameters: TR/TE = 2,000/30 ms, flip angle = 90°, slice thickness/gap = 4/0.4 mm, number of slice = 30, 64 × 64 matrix, field of view = 240 × 240 mm^2^, and acquisition time = 500 s (250 volumes).

### Data Preprocessing

Data preprocessing was performed via Data Processing and Analysis for Brain Imaging (DPABI)^1^ in MATLAB (Yan et al., 2016). The following standard steps were conducted: removing the first ten images, slice timing and motion correction (no participants were excluded due to excessive head motion defined as more than 2 mm translation and/or 2°rotation), normalizing images to the Montreal Neurological Institute (MNI) space and resampling to a 3-mm cubic voxel, smoothing images with a Gaussian kernel of 4-mm full width at half maximum, and temporal filtering in the 0.01–0.08 Hz band, followed by regressing out linear trend, the Friston-24 head motion parameters (Friston et al., 1996), and signals from the white matter and a ventricular region of interest (ROI). We did not perform global signal regression here, as previous studies suggested that the removal of neural signals accompanied with global signals might lead to connectivity distortion (Ben Simon et al., 2017; Glasser et al., 2018).

### Cerebellar Region of Interests Selection and Functional Connectivity Analysis

Three cerebellar seeds involved in the DMN were selected as ROIs: left Crus I (MNI = −33, −76, −34), right Crus I (MNI = 33, −76, −34), and lobule IX (MNI = 0, −55, −49). A 6 mm radius sphere was defined as a ROI for each center point using the REST software (Song et al., 2011). We created correlation maps by calculating Pearson correlation coefficients between the time courses of each ROI and other voxels across the whole brain and then performed Fisher’s *r*-to-*z* transformation to improve the normality of their distribution. Voxel-wise one-way analysis of covariance (ANCOVA) and then *post hoc t*-tests were employed to identify inter-group differences in cerebellar functional connectivity maps with age, sex, years of education, and framewise displacement as covariates. Multiple comparisons were corrected using the Gaussian Random Field (GRF) theory with a significant threshold of *p* < 0.05 (min *z* > 1.96, cluster > 25 voxels).

### Statistical Analysis

Age, years of education, framewise displacement, and clinical characteristics were compared across three groups by using one-way analysis of variance (ANOVA). Sex differences among groups were tested using a chi-square test. The associations of disrupted cerebellar connectivity values with clinical variables in patients with MDD were examined using Pearson correlation analysis. The significant level was set at *p* < 0.05. All the statistical analyses were conducted by using SPSS version 23.

### Classification Analysis Using Support Vector Machine

We selected the significant inter-group differences in the cerebellar-DMN connectivity as features to discriminate patients with MDD from healthy controls and to classify MDD patients with and without GI symptoms using a Gaussian kernel SVM method in the Matlab. Generally, a well-defined sample was used in SVM to create an optimal hyperplane (also called decision boundary) that was capable of distinguishing between categories and predicting a new target subject that belongs to which predefined group (Pereira et al., 2009). We employed a five-fold cross-validation approach here to assess the generalizability of the selected model and avoid overfitting the data. The classifier model was trained using 80% of subjects and then the model performance was evaluated using the remaining data in the “testing phase”. Finally, accuracy, sensitivity, and specificity were computed based on the cross-validation results to quantify the performance of the classifier.

## Results

### Demographic and Clinical Features

As shown in Table 1, the three groups did not differ in age, sex, and years of education. No significant difference was found in illness duration between MDD patients with and without GI symptoms. MDD patients with GI symptoms (G1 group) had higher HRSD-17 scores, higher anxiety/somatization scores, more weight loss, and more sleep disturbances than patients without GI symptoms (G0 group).

**TABLE 1:** Demographic and clinical characteristics of the participants.

	G1 (*n* = 35)	G0 (*n* = 17)	HC (*n* = 28)	*F, t* or χ*^2^* value	*Post hoc t*-tests or *p* values
Age (years)	30.86 ± 6.84	30.29 ± 8.05	30.14 ± 5.00	0.102	0.903^a^
Gender (male/female)	13/22	6/11	14/14	1.377	0.502^b^
Education (years)	14.51 ± 3.28	12.94 ± 3.46	14.61 ± 2.69	1.797	0.173^a^
Illness duration (months)	6.23 ± 4.63	6.94 ± 3.98		0.544	0.589^c^
HRSD-17 scores	22.69 ± 3.41	20.18 ± 2.67	0.89 ± 0.88	585.979	G1 > G0 > HC
Anxiety/somatization	7.31 ± 1.92	6.41 ± 1.66	0.39 ± 0.57	174.531	G1 > G0 > HC
Weight loss	0.80 ± 0.83	0.06 ± 0.24	0	18.741	G1 > G0, HC
Cognitive disturbances	3.71 ± 1.78	3.41 ± 1.50	0	64.213	G1, G0 > HC
Retardation symptoms	6.40 ± 1.42	6.76 ± 1.56	0.18 ± 0.39	253.030	G1, G0 > HC
Sleep disturbances	4.46 ± 1.42	3.53 ± 1.28	0.32 ± 0.55	103.570	G1 > G0 > HC

*G1, MDD patients with gastrointestinal symptoms; G0, MDD patients without gastrointestinal symptoms; HC, healthy controls; HRSD-17, 17-item Hamilton Rating Scale for Depression.*

*^a^The p value was obtained by analyses of variance.*

*^b^The p value was obtained by a chi-square test.*

*^c^The p value was obtained by two-sample t tests.*

### Cerebellar Functional Connectivity Patterns

**FIGURE 1:**
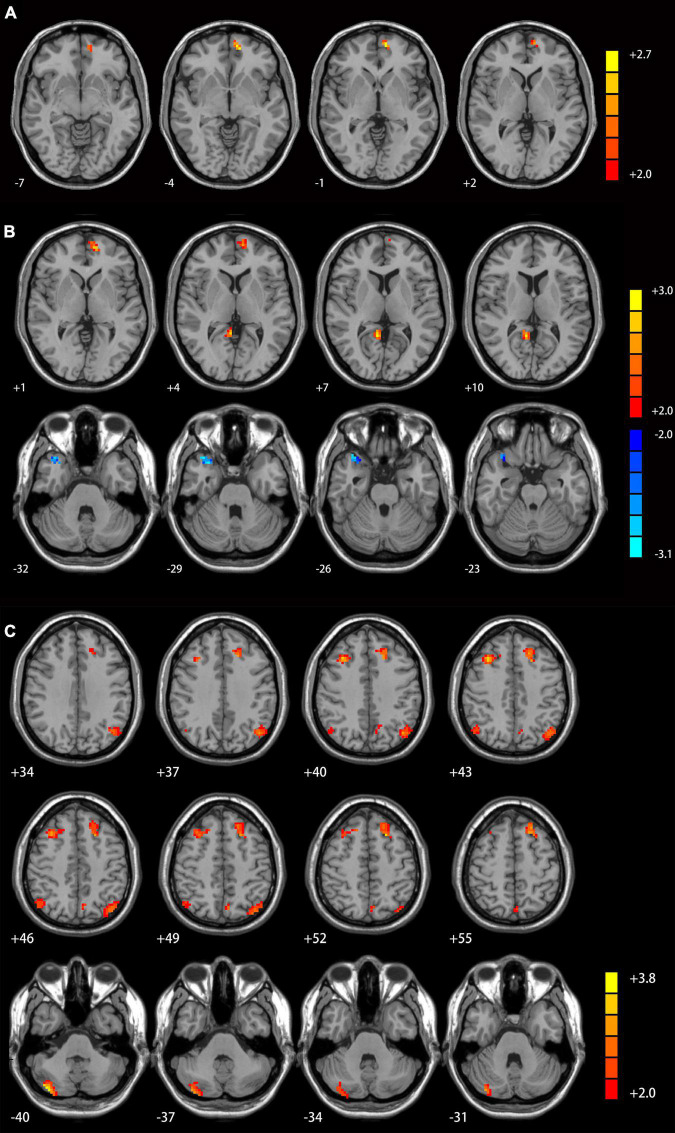
Cerebellar connectivity differences between patients with and without GI symptoms. [**(A)** seed left Crus I; **(B)** seed right Crus I; **(C)** seed lobule IX]. The color bar indicates the *T* values. Results were GRF corrected. GI, gastrointestinal symptoms; GRF, Gaussian random field correction.

Table 2 and Supplementary Table 1 displays significant inter-group differences in the cerebellar functional connectivity patterns of three seed ROIs. Compared with MDD patients without GI symptoms, those with GI symptoms displayed significantly increased functional connectivity between the left Crus I and the left superior medial prefrontal cortex (mPFC; Figure 1A), between the right Crus I and the left superior mPFC and the right posterior cingulate cortex/precuneus (Figure 1B), as well as between the lobule IX and several DMN areas (including the left superior mPFC, bilateral superior frontal gyrus/middle frontal gyrus, bilateral angular gyrus, left precunues, and right cerebellum Crus I/II, Figure 1C). Decreased functional connectivity was found between the right Crus I and the right precunues (Figure 1B).

**TABLE 2:** Cerebellar functional connectivity differences across the participants.

Cluster location	Peak (MNI)			Number of voxels	*T* value
	*x*	*y*	*z*		
**Seed: left crus I**					
**G1 vs G0**					
Left superior mPFC	−9	57	−3	29	2.6298
**G1 vs HC**					
Bilateral precuneus	9	−72	27	137	3.4230
Right inferior parietal lobule	42	−60	42	68	2.9813
Left superior temporal gyrus	−36	15	−27	39	−4.5190
Left middle temporal gyrus	−51	−27	−6	42	−3.1027
Left inferior temporal gyrus	−51	−9	−30	40	−3.1181
Right middle temporal gyrus	45	−3	−27	49	−3.1517
**Seed: right crus I**					
**G1 vs G0**					
Left superior mPFC	−12	54	−3	40	2.6852
Right posterior cingulate cortex/precuneus	9	−51	6	59	2.8385
Right precuneus	39	21	−30	46	−2.8312
**G1 vs HC**					
Right cerebellum crus II	30	−84	−42	26	3.5445
Right middle temporal gyrus/inferior temporal gyrus	51	6	−24	141	−2.9582
Bilateral mPFC/anterior cingulate cortex	12	45	−9	136	−2.9683
**Seed: lobule IX**					
**G1 vs G0**					
Left superior mPFC	−21	60	3	81	3.4249
Left superior frontal gyrus/middle frontal gyrus	−21	21	51	153	3.4111
Right superior frontal gyrus/middle frontal gyrus	33	24	42	89	3.3205
Left angular gyrus	−45	−69	36	139	2.9175
Right angular gyrus	51	−69	45	42	2.5483
Left precuneus	−6	−72	48	26	2.7776
Right cerebellum crus I/II	39	−81	−39	83	3.8131
**G1 vs HC**					
Right middle frontal gyrus	36	27	48	48	3.1125
Right precuneus	9	−69	24	28	3.0119

*G1, MDD patients with gastrointestinal symptoms; G0, MDD patients without gastrointestinal symptoms; HC, healthy controls; MNI, Montreal Neurological Institute; mPFC, medial prefrontal cortex.*

**FIGURE 2:**
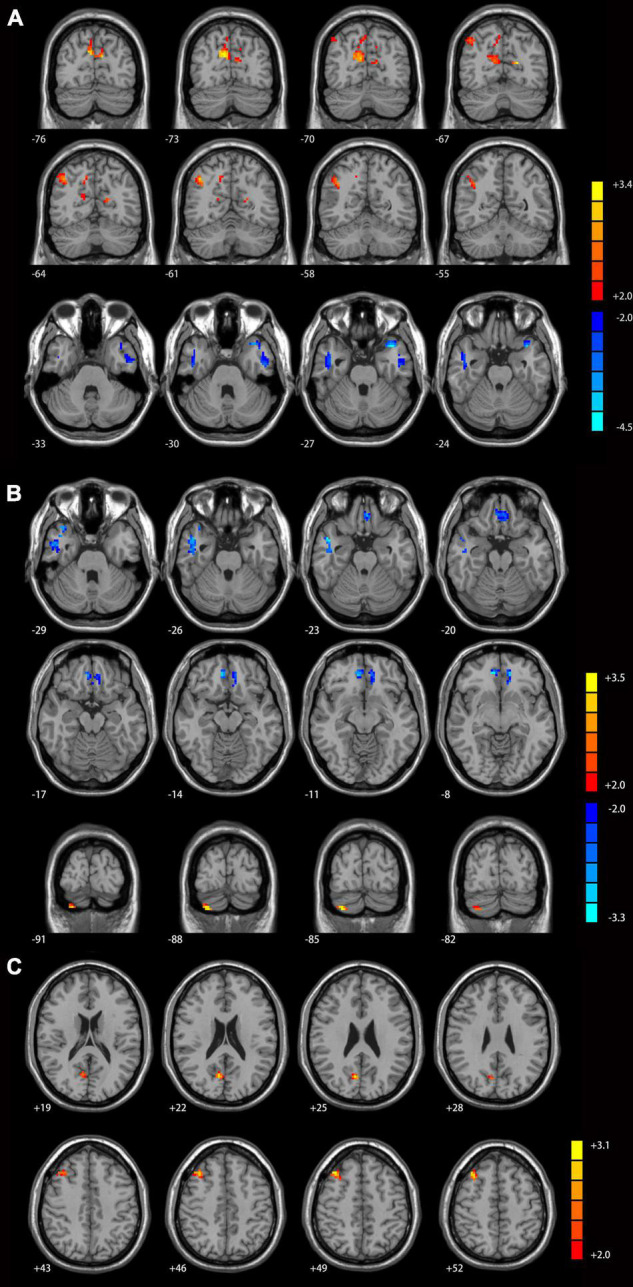
Cerebellar connectivity differences between major depressive disorder (MDD) patients with GI symptoms and healthy controls [**(A)** seed left Crus I; **(B)** seed right Crus I; **(C)** seed lobule IX]. The color bar indicates the *T* values. Results were GRF corrected. GI, gastrointestinal symptoms; GRF, Gaussian random field correction.

Compared with healthy controls, MDD patients with GI symptoms had increased functional connectivity between the left Crus I and bilateral precunues and right inferior parietal lobule (Figure 2A), between the right Crus I and right cerebellum Crus II (Figure 2B), as well as between the lobule IX and right middle frontal gyrus and right precunues (Figure 2C). Decreased functional connectivities were observed between the left Crus I and lateral temporal cortex (including the left superior and inferior temporal gyrus and bilateral middle temporal gyrus, Figure 2A), as well as between the right Crus I and right middle temporal gyrus/inferior temporal gyrus and bilateral mPFC/anterior cingulate cortex (Figure 2B).

### Correlations With Clinical Variables

**FIGURE 3:**
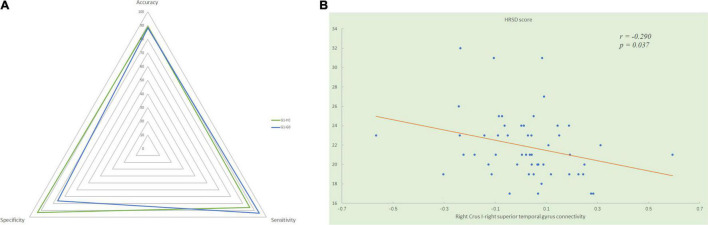
**(A)** Significantly negative correlation between the HRSD scores and the mean functional connectivity values of the right Crus I - right superior temporal gyrus connectivity in all patients with MDD. **(B)** The radar graph shows the accuracy, sensitivity, specificity of the classifications of group G1 versus G0 and group G1 versus HC. HRSD, Hamilton Rating Scale for Depression; G1, MDD patients with gastrointestinal symptoms; G0, MDD patients without gastrointestinal symptoms; HC, healthy controls.

We extracted the mean functional connectivity values of the significant inter-group differences and examined whether any of them were associated with clinical variables. In all patients with MDD, functional connectivity between the right Crus I and the right superior temporal gyrus was negatively correlated with GI symptoms scores (*r* = −0.378, *p* = 0.006) and HRSD scores (*r* = −0.290, *p* = 0.037, Figure 3A). Significant positive correlations were found between the GI symptoms scores and the values of the following connectivity: the right Crus I-left superior mPFC (*r* = 0.299, *p* = 0.031), the right Crus I-right precunues (*r* = 0.381, *p* = 0.005), lobule IX-left angular (*r* = 0.277, *p* = 0.047), lobule IX-right angular (*r* = 0.308, *p* = 0.026), lobule IX-right Crus I (*r* = 0.495, *p* < 0.000), lobule IX-left superior frontal gyrus (*r* = 0.376, *p* = 0.006), and lobule IX-right superior frontal gyrus (*r* = 0.352, *p* = 0.011).

### Discrimination Between Major Depressive Disorder Patients With Gastrointestinal Symptoms and Healthy Controls

Using significantly disrupted cerebellar-DMN connectivities derived from the seed-based functional connectivity analysis between MDD patients with GI symptoms and healthy controls as classification features, we performed SVM analysis and found they could achieve a satisfactory discriminate rate of 89% (56 of 63 in the two groups), with a sensitivity of 86% and a specificity of 93%. Similarly, the between-group differences of cerebellar-DMN circuits in G1–G0 comparison could correctly classify depressed patients with GI symptoms into G1 group with an accuracy of 88% (46 of 52 in the two groups), a sensitivity of 94%, and a specificity of 76% (Figure 3B).

## Discussion

This study focused on GI symptoms in first-episode, drug-naive patients with MDD and preliminarily explored the resting-state cerebellar functional connectivity patterns of these patients. First, consistent with previous studies, more severe depressive symptoms were found in patients with GI symptoms in the psychiatric care settings compared with those without GI symptoms and healthy controls. Our results demonstrated that patients with GI symptoms have more severe anxiety/somatization symptoms, greater weight loss, and more severe sleep disturbances than patients without GI symptoms and healthy controls. Second, significantly increased cerebellar-anterior DMN connectivities were found in patients with MDD accompanied with GI symptoms compared with those without GI symptoms. Both increased and decreased functional connectivities were found between cerebellum and posterior DMN in patients with GI symptoms compared with those without GI symptoms and healthy controls. Moreover, our results revealed that the right Crus I - right superior temporal gyrus connectivity value was related to severity of GI symptoms and depression in all patients with MDD. Third, our SVM analysis further demonstrated a satisfactory classification accuracy (89%) of these disrupted cerebellar connectivities for correctly identifying patients with GI symptoms, which indicated the potential role of cerebellar-DMN circuits in the mechanisms of MDD with co-occurring GI symptoms.

As expected, we found that depressed patients with GI symptoms experienced more severe depression and anxiety/somatization. Similar results have also been reported by studies focusing on patients with FGIDs. Compared with patients without FGIDs, prevalence of depression or anxiety was increased in patients with FGIDs, and among them, asymptomatic patients had the lowest prevalence. In addition, the increase of the prevalence of depression and anxiety exhibited in a stepwise manner with the increase of the frequency and/or severity of GI symptoms (Pinto-Sanchez et al., 2015). The close relationship between GI symptoms and the depression or anxiety may indicate the common pathophysiological mechanisms behind them. For example, the bidirectional communication between brain and gut (brain-gut interaction) is one of the fields received much attention. The important role gut microbiota play in psychosomatic disorders and psychiatric illnesses have been suggested in a growing body of studies (Labus et al., 2017; Strandwitz et al., 2019). Some neuroimaging studies have explored the effects of gut microbiota on the pattern of emotional response to negative stress stimuli. Bagga et al. (2018) found an increased accuracy for unpleasant pictures in an emotional recognition memory task in healthy subjects who received probiotics intervention, and simultaneously reduced cerebellar activity was observed during the task. Decreased functional connectivity in the DMN was observed in the resting state after intervention (Bagga et al., 2019). A significantly negative association between the relative abundance levels of γ-aminobutyric acid (GABA)-producing bacteria in feces and functional connectivity in the DMN was reported in patients with MDD (Strandwitz et al., 2019). These findings highlight the importance of GI symptoms in the diagnosis and treatment of MDD. Probiotic-based therapies for GI symptoms in MDD are promising but more researches are needed to elucidate the brain-gut pathways.

The DMN is a critical large-scale network and encompasses two subsystems (the anterior and posterior part) contributing differently to the pathopsychological characteristics of MDD (Xu et al., 2016). The mPFC/ACC and posterior cingulate cortex (PCC)/precunues are hub areas of the two subsystems, respectively. Increased functional connectivity in mPFC and ventral ACC were reported in patients with MDD by Zhu et al. (2012) by using independent component analysis, and the functional connectivity values of the two regions were positively associated with rumination scores, while decreased functional connectivity was observed in the PCC/precuneus and bilateral angular gyrus, and their values were negatively associated with overgeneral autobiographical memory. Relative to healthy controls, decreased cerebellar-mPFC functional connectivity was observed in patients with treatment-sensitive depression and treatment-resistant depression in our previous study (Guo et al., 2013). Similarly, compared with healthy controls, reduced cerebellar-ventral mPFC dynamic functional connectivity was found in patients with MDD (Zhu et al., 2020). Consistent with these studies, we found decreased cerebellar-anterior DMN connectivity in depressed patients with GI symptoms when compared with healthy controls. While compared with depressed patients without GI symptoms, increased cerebellar-anterior DMN connectivity was found in those with GI symptoms. Moreover, the functional connectivity values of several regions within DMN were positively associated with GI symptoms. Interestingly, the mPFC and extended regions in the caudolateral orbital cortex (including regions in PCC and superior temporal gyrus) form the medial prefrontal network, which is prominently related to limbic areas and visceral control areas (hypothalamus and periaqueductal gray), and is considered as the visceromotor system engaging in introspective functions and visceral reactions to emotional stimuli (Ongur and Price, 2000; Drevets et al., 2008). Our study adds further evidence for the abnormal cerebellar-DMN circuits in MDD from a brain-gut perspective. Especially, the mPFC may play a key role in not only the emotion processing but also the GI responses in depression.

Aberrant serotonin (5-HT) function have been found in diverse cerebral regions (including mPFC/ACC) in MDD (Savitz et al., 2009), and these abnormalities are suggested to be contributors to the development and modulation of somatic symptoms (Liu Y. et al., 2019). Antidepressants such as selective 5-HT reuptake inhibitors and dual 5-HT and norepinephrine reuptake inhibitors are effective in treating somatic complaints in depression and FGIDs (Jackson et al., 2000). One of the possible underlying mechanisms may be their 5-HT modulating effect in mPFC/ACC. Lopez-Sola et al. (2010) found that clinical improvement after duloxetine treatment in patients with MDD was associated with pain-related activation alterations in ACC and prefrontal cortex. In addition, the serotonergic fiber is the third largest population of various amines and peptides input received by the cerebellum (Schweighofer et al., 2004). Cerebellar serotonergic system has been related to motor activity (Kawashima, 2018), and previous studies have suggested an association between cerebellar serotonin dysfunction and cerebellar ataxia (Ogawa, 2004). The linkage between this system and cognitive process remains unclear. Our findings provided preliminary evidence of possible modulatory effects of 5-HT in cerebellar-DMN circuits for somatic symptoms in MDD.

The lateral temporal gyrus, parts of DMN, is one of the well-acknowledged areas involved in the pathophysiology of MDD. They play crucial roles in social cognition and emotion regulation (Schaefer et al., 2006; Goulden et al., 2012). A great number of studies have reported morphologic changes in these regions. For example, relative to healthy controls or remitter patients with MDD, significant volume and cortical thickness reduction were found in the superior and middle temporal gyrus in patients with acute MDD (Takahashi et al., 2010; Saricicek Aydogan et al., 2019; Li et al., 2020). Functional abnormalities of the lateral temporal gyrus in MDD were also supported by fMRI studies. Enhanced regional homogeneity has been observed in the superior, middle and inferior temporal gyrus in treatment-refractory depression (Guo et al., 2011; Wu et al., 2011). In contrast, we found decreased functional connectivity between cerebellum and lateral temporal gyrus in first-episode drug-naïve patients with MDD regardless of GI symptoms, and a negative correlation was found between the functional connectivity value of the cerebellar-superior temporal gyrus and HRSD scores. Decreased connections in the superior temporal gyrus for functional network was also reported in patients with MDD by using network-based statistic approach (Yao et al., 2019). Taken together, our study and previous studies reveal that the lateral temporal part of DMN is related to the pathology of MDD.

There are still several limitations in this study. First, the small sample size may affect the reliability of our results. Second, the severity of GI symptoms was evaluated by only one item from the HRSD. Although some previous studies (Liu et al., 2020; Liu P. H. et al., 2021) also employed the same method, it is too rough and may affect the results. Future studies in a larger sample and using specific scales for GI symptoms are needed to validate our findings. Third, it is unknown that whether the findings could generate to patients who visited general physicians since we recruited depressed patients exclusively from psychiatric care settings. For example, given that most patients with MDD tend to visit physicians first instead of psychiatric professionals, it is possible that depressed patients with GI symptoms who visited general physicians have milder depressive symptoms than those go to psychiatric care settings. Fourth, we adopted a relatively lenient correction method and threshold, so we cannot claim that the type I error was strongly controlled. Finally, given the possibility of comorbidity and overlapping of symptoms, it is as valuable to differentiate between other psychiatric disorders (such as anxiety disorders, bipolar disorder and posttraumatic stress disorder) and MDD as identifying MDD from healthy controls. Future studies should recruit patients with other psychiatric disorders and complement the current findings by exploring resting-state fMRI features for differential diagnosis.

In conclusion, abnormal cerebellar-DMN functional connectivity was observed in patients with MDD accompanied with GI symptoms. The high classification results between depressed patients with GI symptoms and those without GI symptoms and healthy controls obtained by SVM analysis revealed the possible neural mechanisms for the involvement of cerebellar-DMN circuits in GI symptoms co-occurred with MDD.

## Data Availability Statement

The raw data supporting the conclusions of this article will be made available by the authors, without undue reservation.

## Ethics Statement

The studies involving human participants were reviewed and approved by the Medical Research Ethics Committee of the Second Xiangya Hospital of Central South University, China. The patients/participants provided their written informed consent to participate in this study.

## Author Contributions

All authors contributed to the manuscript and approved the final version.

## Conflict of Interest

The authors declare that the research was conducted in the absence of any commercial or financial relationships that could be construed as a potential conflict of interest.

## Publisher’s Note

All claims expressed in this article are solely those of the authors and do not necessarily represent those of their affiliated organizations, or those of the publisher, the editors and the reviewers. Any product that may be evaluated in this article, or claim that may be made by its manufacturer, is not guaranteed or endorsed by the publisher.

## Footnotes

1
http://rfmri.org/dpabi
