# Supplementary material for: Disrupted Cerebellar-Default Mode Network Functional Connectivity in Major Depressive Disorder With Gastrointestinal Symptoms
Source: Front Cell Neurosci. 2022 Mar 3;16:833592. doi: 10.3389/fncel.2022.833592 (PMC8927069; doi:10.3389/fncel.2022.833592)
Supplement: Supplementary file 1 [file Table_1.DOCX]

Table S1 Cerebellar functional connectivity differences across the participants.

| Cluster location | Peak (MNI) | | | Number of voxels | *T* value |
| --- | --- | --- | --- | --- | --- |
|  | x | y | z |  |  |
| *Seed: Left Crus I* |  |  |  |  |  |
| *G0 vs HC* |  |  |  |  |  |
| Right Precuneus | 6 | -75 | 45 | 58 | 2.7888 |
| Right Angular Gyrus | 39 | -54 | 33 | 26 | 3.3350 |
| Right Middle Cingulate Gyrus | 15 | -39 | 36 | 47 | 3.2255 |
| Right Cerebellum Crus1 | 21 | -75 | -33 | 53 | -2.5274 |
| Left Superior mPFC | -21 | 60 | 27 | 248 | -3.2630 |
| Left Superior Temporal Gyrus | -48 | -54 | 24 | 88 | -2.7471 |
|  |  |  |  |  |  |
| *Seed: Right Crus I* |  |  |  |  |  |
| *G0 vs HC* |  |  |  |  |  |
| Right mPFC | 6 | 51 | -9 | 72 | -2.6545 |
| Left mPFC | -15 | 42 | -12 | 53 | -3.1646 |
| Right Posterior Cingulate Cortex/Precuneus | 6 | -45 | 3 | 63 | -2.7511 |
| Left Precuneus | -30 | -81 | 39 | 36 | -3.2327 |
| Right Superior Frontal Gyrus/Middle Frontal Gyrus | 24 | 24 | 39 | 136 | -3.5548 |
| Left Superior Frontal Gyrus/Middle Frontal Gyrus | -24 | 33 | 45 | 43 | -3.6085 |
|  |  |  |  |  |  |
| *Seed: Lobule IX* |  |  |  |  |  |
| *G0 vs HC* |  |  |  |  |  |
| Left Superior Frontal Gyrus | -21 | 54 | 9 | 26 | -2.8009 |
| Left Superior Frontal Gyrus/Middle Frontal Gyrus | -18 | 24 | 39 | 143 | -3.8537 |
| Right Superior Frontal Gyrus | 21 | 45 | 45 | 44 | -2.5534 |
| Left Angular Gyrus | -48 | -75 | 33 | 231 | -2.9286 |
| Bilateral Middle Cingulate Gyrus | 0 | -24 | 33 | 29 | -2.9087 |
| Right Cerebellum Crus1/2 | 36 | -84 | -30 | 38 | -2.9203 |

Abbreviations: G0, MDD patients without gastrointestinal symptoms; HC, healthy controls; MNI, Montreal Neurological Institute; mPFC, medial prefrontal cortex.
